# Supplementary material for: Data-driven design of electrolyte additives supporting high-performance 5 V LiNi0.5Mn1.5O4 positive electrodes
Source: Nat Commun. 2025 Apr 10;16:3413. doi: 10.1038/s41467-025-57961-w (PMC11986164; doi:10.1038/s41467-025-57961-w)
Supplement: Supplementary file 1 — Supplementary Information [file 41467_2025_57961_MOESM1_ESM.pdf]

Supplementary Information

Data-driven Design of Electrolyte Additives Supporting High-Performance 5 V $LiNi_{0.5}Mn_{1.5}O_4$ Positive Electrodes

Bingning Wang,[1,†] Hieu A. Doan,[2,†] Seoung-bum Son,[1] Daniel P. Abraham,[1] Stephen E. Trask,[1] Andrew Jansen,[1] Kang Xu,[3,*] Chen Liao[1,4,*]

[1]Chemical Sciences and Engineering Division, Argonne National Laboratory, 9700 South Cass Avenue, Lemont, Illinois, 60439, United States

[2]Materials Science Division, Argonne National Laboratory, 9700 South Cass Avenue, Lemont, Illinois, 60439, United States

[3]SES AI Corps, 35 Cabot Road, Woburn, MA, 01801, United States

[4]Energy Storage Research Alliance, Argonne National Laboratory, 9700 South Cass Avenue, Lemont, IL 60439, USA

[†]These authors contributed equally: Bingning Wang and Hieu A. Doan

*Corresponding authors: Kang Xu, kang.xu@ses.ai; Chen Liao, liaoc@anl.gov

Contents

Supplementary Note 1. Design of the protocol........ 3

Supplementary Note 2. Laminate optimization. 4

Supplementary Note 3. Electrochemical Performance including Cycling and Impedance........ 7

Supplementary Note 4. Justification of feature set selection 13

Supplementary Note 5. Benchmark of ML algorithms........ 17

Supplementary Note 6. Justification of the use of machine learning (ML) for 125 unknown additives........ 23

Supplementary Note 7. Selection of predicted additive candidates via Exploitation and Bayesian optimization........ 24

Supplementary Note 8. Capacity Loss Mechanisms demonstrated by various methods 27

8.1 Diagnostic Cycling of the Harvested Cells 27

8.2 ^{1}H Nuclear Magnetic Resonance 31

8.3 X-ray Photoelectron Spectroscopy (XPS) 32

8.4 Inductively Coupled Plasma Mass Spectrometry 34

8.5 Scanning Electron Microscopy and Energy-dispersive X-ray spectroscopy (EDX) analysis 35

8.6 Online Electrochemical Mass Spectrometry (OEMS) 42

Supplementary References 44

Supplementary Note 1. Design of the protocol

The design of the protocol of Gr||$LiNi_{0.5}Mn_{1.5}O_4$ (Gr||LNMO) is critical for identification of decomposition products and development of viable additives for improvements. A modification was made to the previous protocol[1] in order to reduce the duration of one complete cycle, which currently spans approximately 60 days. The revised protocol comprises the following steps:

a. Formation step of 5 cycles at C/10 cycles For LNMO positive electrodes, using a standard active weight of 14.46 mg, the current used for 1C rate is 1.74 mA, assuming the theoretical capacity for LNMO is 120 mAh g^{-1}. C/10 is 174 µA.

b. Reference performance test (RPT) of C/25. Note Please note that the revised protocol incorporates a slower rate compared to our previous results on the $LiNi_{0.9}Mn_{0.05}Co_{0.05}O_2$ study.[2] This slower RPT is specifically designed to counteract the impedance effect observed at higher C rates, while simultaneously ensuring the generation of distinct and well-defined voltage plateaus. For LNMO positive electrodes, using a standard active weight of 14.46 mg, the current used for 1C rate is 1.74 mA, assuming the theoretical capacity for LNMO is 120 mAh g^{-1}. C/25 is 69 µA.

c. Preparation cycle of 1/C and one Hybrid Pulse Power Characterization (HPPC) cycle.[3] The HPPC cycle is composed of 10-second pulses of 2C discharge and 1.5C charge. The currents in this context are expressed as C-rates, where 1C is approximately 1.7 mA.

d. Aging cycles at a rate of C/3, the upper cutoff voltage is 4.7 V with 1 h hold (current drop to C/20) and a lower cutoff voltage of 3.8 V. Due to the relatively high voltage of 4.7 V, observable delamination is more prominently manifested in the large

format (Φ=20 cm^2) cells rather than the coin cells (Φ=1.54 cm^2), In order to assess the performance of both the baseline and the best performers, we have made coin cells using Armor-SAS polymer and carbon-coated Al current collectors, while keeping all other parameters unchanged. This choice of current collector is aimed at addressing the delamination issue and evaluating the performance of the cells. C/20 is 87 µA.

e. Repeat steps b-d for a total of 4 loops

f. The last RPT cycle of C/25 followed by one HPPC cycle.

Supplementary Note 2. Laminate optimization.

Unlike the previous NMC (Nickel Manganese Cobalt) positive electrode

materials, the LNMO (Lithium Nickel Manganese Oxide) positive electrodes exhibited delamination issues that were further exacerbated in larger formats.[4] In the current testing, where coin cell format with a 14 mm diameter positive electrode was utilized, the delamination had minimal impact on the cycling performance, allowing for good repeatability of results. One strategy to mitigate the delamination is to use specialty current collector, for example, a polymer-coated aluminum current collector provided by ARMOR-SAS.[5] However, a noticeable discrepancy was observed in the baseline electrolyte system for LNMO laminates using ARMOR-SAS. As shown in Supplementary Fig. 1, in comparison to the non-coated laminates (represented by black circles), the coated laminates (represented by black squares) demonstrated a more inferior cycling performance. This indicates that the ARMOR-SAS polymer coating may have had a negative impact on the cycling behavior of the LNMO laminates. Further investigation is required to understand the underlying factors contributing to this discrepancy and identify potential solutions and will be reported in the future.

Nevertheless, when additives were introduced to the system, a similar performance was observed between the coated and non-coated systems when 0.25 wt% tVCBO + 1 wt% MS or 1 wt% LiDFOB + 1 wt% TMSPi was used. This suggests that the non-coated laminate does not interfere with the efficacy of additives. In fact, all non-coated laminate performs better in presence of the additives then their coated laminate counterparts. The results indicate that the potential high voltage, which could potentially cause delamination, does not emerge as a primary issue that disrupts the

evaluation of additive efficiency. This comparison emphasizes that the presence of the non-coated laminate does not significantly affect the results and validity of assessing the effectiveness of the additives. (Supplementary Fig. 1)

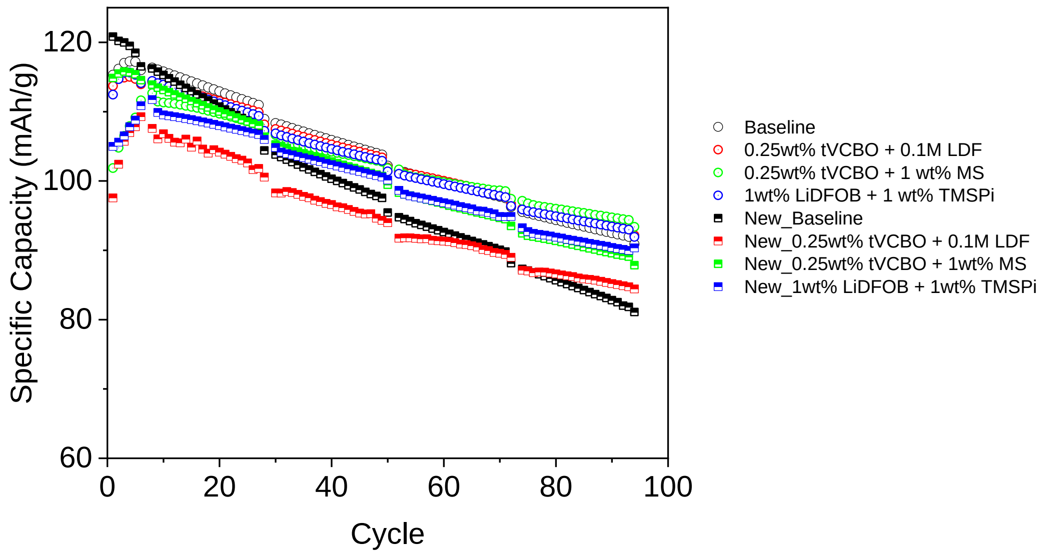

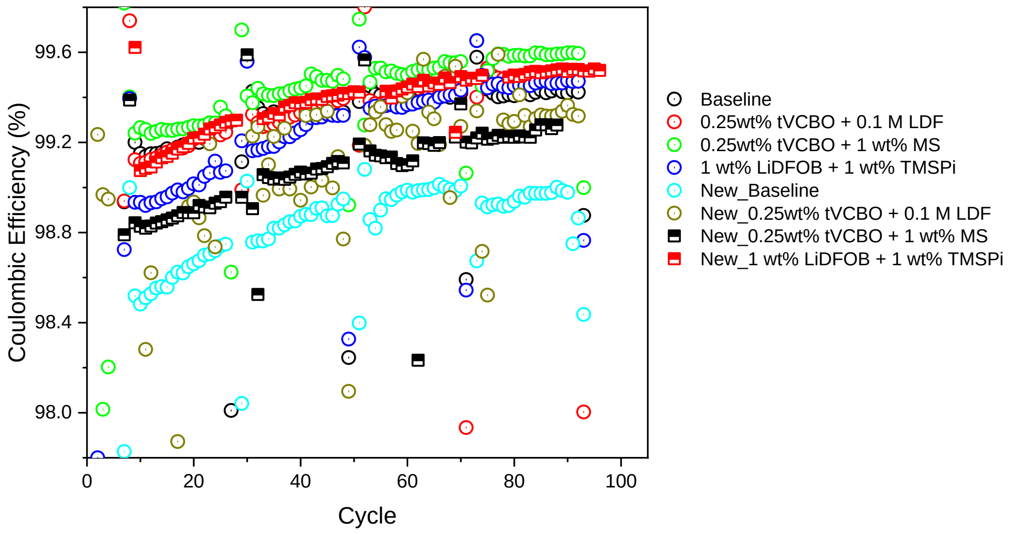

Supplementary Figure 1. Electrochemistry of the LNMO laminates in the absence and presence of a polymer coating on the current collector. Top: Comparison of specific capacities between old and new LNMO laminates. The symbols in circles represent laminates w/o Armor- SAS polymer, while symbols in squares represent new laminates with SAS Armor Polymer. Electrolytes with additives show good performance for both the laminates with and without Armor Polymer. Bottom: Comparison between old and new LNMO of their coulombic efficiency.

Supplementary Note 3. Electrochemical Performance including Cycling and Impedance

The overall electrochemical performance of the additives is presented in Supplementary Fig. 2. The baseline electrolyte comprises 1.0 M of $LiPF_6$ in EC/EMC (1/9) with a lower EC content compared to previous generations, which were utilized for other transition metal oxide cathodes (TMOs). The reduced EC content is attributed to its ring structure, which exhibits lower anodic stability than other carbonates.[14] According to Manthiram et al, [15] a lower EC content is preferable for high voltage cathodes/positive electrodes to minimize side reactions at high states of charge (SOC).

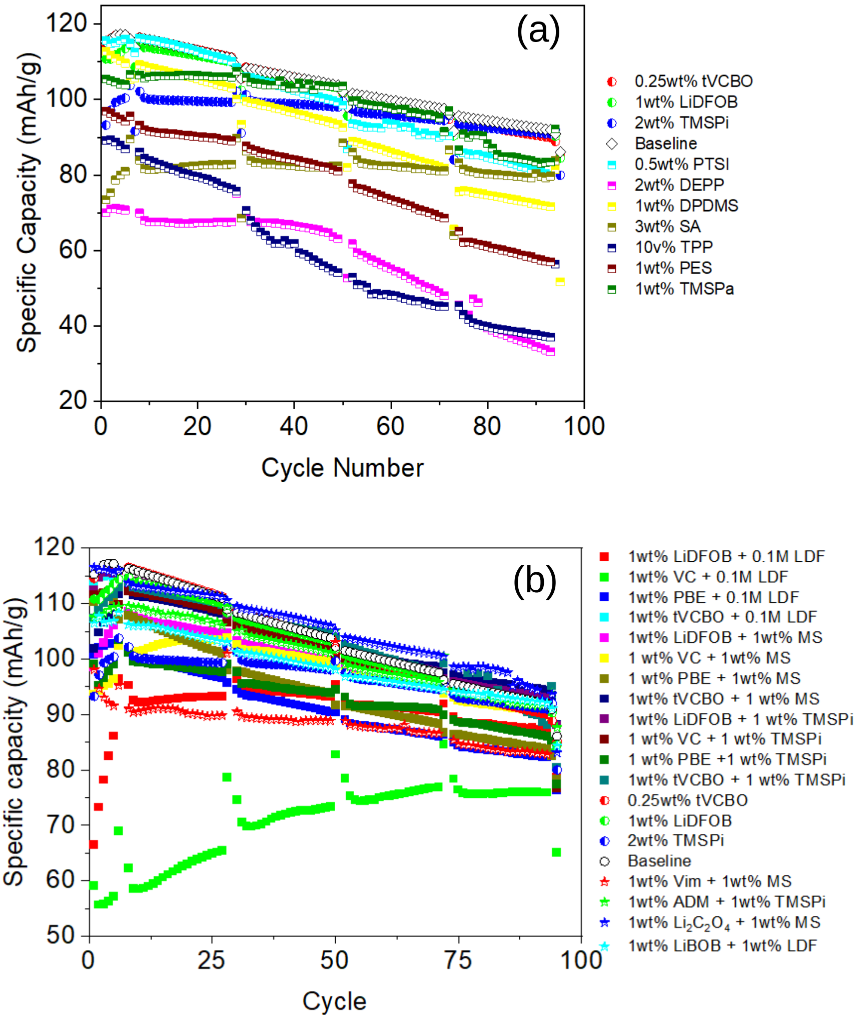

(a)
Specific Capacity (mAh/g)
Cycle Number
0.25wt% tVCBO
1wt% LiDFOB
2wt% TMSPi
Baseline
0.5wt% PTSI
2wt% DEPP
1wt% DPDMS
3wt% SA
10v% TPP
1wt% PES
1wt% TMSPa
(b)
Specific capacity (mAh/g)
Cycle
1wt% LiDFOB + 0.1M LDF
1wt% VC + 0.1M LDF
1wt% PBE + 0.1M LDF
1wt% tVCBO + 0.1M LDF
1wt% LiDFOB + 1wt% MS
1 wt% VC + 1wt% MS
1 wt% PBE + 1wt% MS
1wt% tVCBO + 1 wt% MS
1wt% LiDFOB + 1 wt% TMSPi
1 wt% VC + 1 wt% TMSPi
1 wt% PBE +1 wt% TMSPi
1wt% tVCBO + 1 wt% TMSPi
0.25wt% tVCBO
1wt% LiDFOB
2wt% TMSPi
Baseline
1wt% Vim + 1wt% MS
1wt% ADM + 1wt% TMSPi
1wt% $Li_2C_2O_4$ + 1wt% MS
1wt% LiBOB + 1wt% LDF

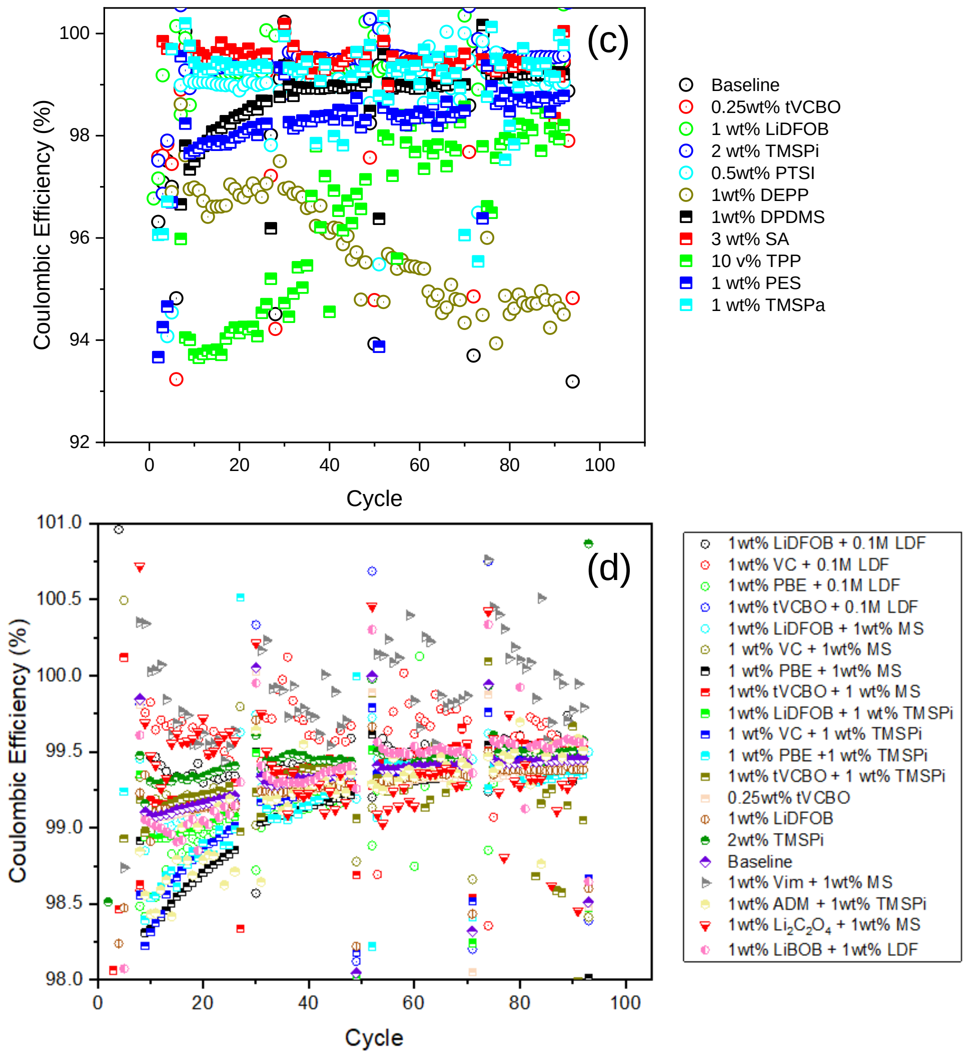

Supplementary Figure 2. Analysis of electrochemical performance. Specific capacities of Gr||LNMO using (a) single additives (b) dual additives. Coulombic efficiencies of Gr||LNMO using (c) single additives (d) dual additives. The data represents the average of three cells for each formulation, and the coin cells were cycled at 30 oC with the protocol mentioned in Supplementary Note 1.

The HPPC (Hybrid Pulse Power Characterization) data of the additives is presented in Supplementary Fig. 3. Specifically, additives containing LiDFOB exhibit the best performance in terms of reducing the initial Area Specific Impedance (ASI) and inhibiting its increase during cycling.

In a parallel study, it was observed that the main impedance in the Gr||LNMO

(graphite) system originates from the positive electrode side, and the composition of the laminate significantly impacts the impedance. For instance, the addition of 0.05 wt% Single-Wall Carbon Nanotube (SWCNT)in the slurry composition for the laminate resulted in a reduction of the initial impedance from 55 Ω·cm^2 to 25.58 Ω·cm^2 (unpublished data).

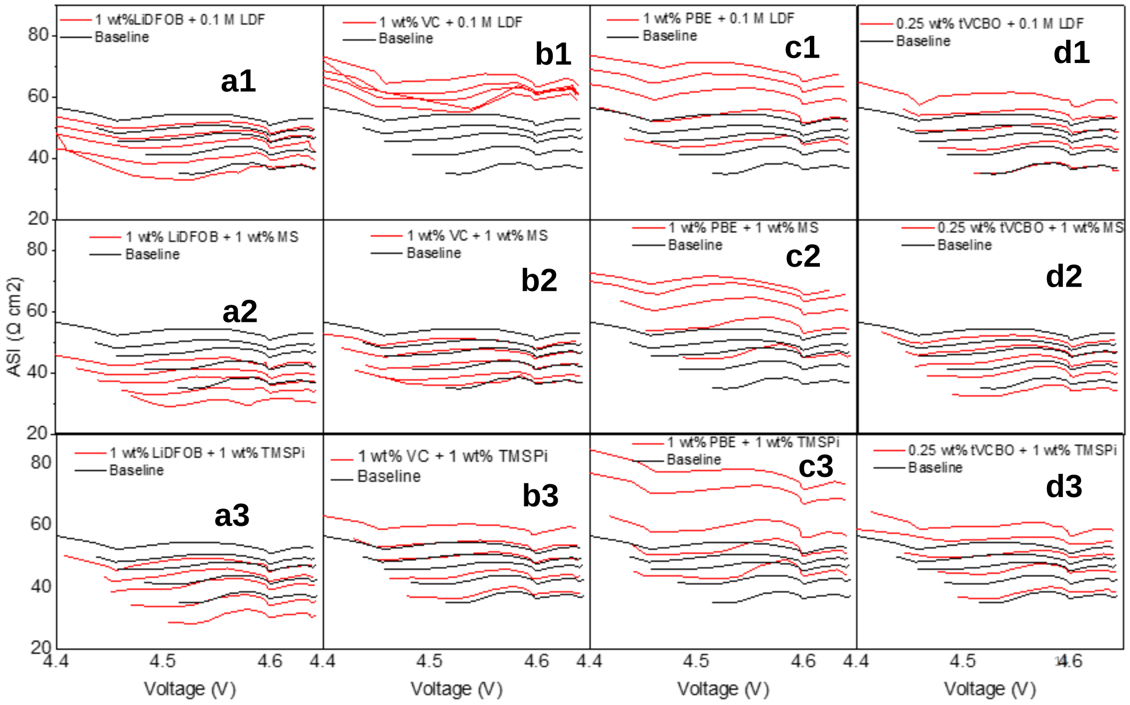

Supplementary Figure 3. HPPC (Hybrid Pulse Power Characterization) data of additives. The HPPC was measured at 9, 32, 55, 78, 101 cycle. Two additives demonstrate superior performance compared to the baseline electrolyte of 1.0 M LiPF6 in EC/EMC (1/9): 1 wt% LiDFOB + 1 wt% TMSPi and 0.25 wt% tVCBO + 1 wt% MS. Specifically, additives containing LiDFOB (a1-3) exhibit the best performance in terms of reducing the initial area specific Impedance (ASI) and inhibiting its increase during cycling.

The final ASI, the impedance rise (ΔASI), as well as the final specific capacity (Q) in the experimentally validated results are shown in Supplementary Table 1.

Supplementary Table 1 Summary of ASI, ∆ASI, and Q for experimentally tested additives. The ASI and ∆ASI were measure at 4.5 V. The final Q is taken from cycle 99.

Electrolyte Composition	Final ASI (Ω•cm^2)	Delta ASI	Final Capacity
0.25wt% tVCBO	50.15	18	89.966
1wt% LiDFOB	45.78	17	91.235
2wt% TMSPi	50.29	16	90.96
0.5wt% PTSI	69.53	41	79.961
2wt% DEPP	143.07	88	33.216
1wt% DPDMS	72.92	39	71.673
3wt% SA	81.72	24	79.728
10wt% TPP	105.10	57	37.072
1wt% PES	118.84	76	56.982
1wt% TMSPa	50.99	17	83.502
1wt% LiDFOB + 0.1M LDF	50.79	17	87.057
1wt% VC + 0.1M LDF	65.58	7	75.883
1wt% PBE + 0.1M LDF	71.36	28	82.240
0.25wt% tVCBO + 0.1M LDF	61.18	26	93.334
1wt% LiDFOB + 1wt% MS	44.21	15	90.903
1wt% VC + 1wt% MS	51.01	15	90.196
1 wt% PBE +1 wt% MS	71.76	27	83.830
0.25 wt% tVCBO + 1 wt% MS	51.26	19	94.367
1wt% LiDFOB + 1wt% TMSPi	49.01	21	92.980
1wt% VC + 1wt% TMSPi	60.01	23	92.011
1 wt% PBE +1 wt% TMSPi	77.95	35	86.013
0.25wt% tVCBO + 1 wt% TMSPi	59.62	23	88.058
1wt% $Li_2C_2O_4$ + 1wt% MS	60.09	19	91.579
1wt% LiBOB + 1wt% LDF	54.77	16	92.042
1wt% ADM + 1wt% $LiOtC_4F_9$	118.40	80	42.188
1wt% LiBOB + 1wt% LiBFEP	180.22	94	50.021
1wt% LiDFOB + 1wt% LiBFEP	111.47	53	66.744
1wt% ADM + 1wt% TMSPi	129.23	46	91.470
Baseline	54.47	19	91.942

1wt% LiDFOB + 1wt% SA	41.19	16	91.792
1wt% LiBOB + 1wt% TMSPi	104.76	66	69.803
1wt% LiBOB + 1wt% MS	50.13	17	92.171
1wt% LiBOB + 1wt% SA	46.79	10	95.486
1wt% VC + 1wt% SA	123.53	86	47.072
1wt% LiBOB + 1wt% LDF	203.43	110	80.061

Supplementary Note 4. Justification of feature set selection

To improve the clarity of our feature engineering process, we have benchmarked the accuracy of GPR models using additional feature sets, with the results presented in Supplementary Table 2. The physicochemical feature set comprises 66 unique physical and chemical descriptors, including molecular weight, topological polar surface area, number of valence electrons, and number of aromatic rings. The extended connectivity fingerprint (ECFP) is a representation based on atom-centered circular neighborhoods, where atom environments are iteratively expanded and hashed into fixed-length binary vectors. Both the physicochemical features and ECFP can be automatically generated for each additive system using the RDKit package.[20] As shown in Supplementary Table 2, models employing either ECFP or the unique atom and coordination count feature set exhibit significantly higher accuracy compared to those using physicochemical features. Although ECFP offers a slight performance improvement over the unique atom and coordination count feature set, its large number of features and lack of interpretability make it less suitable for a small dataset (i.e., 28 experimental measurements). Therefore, we believe that our chosen feature set-counting unique atoms and their coordination-provides the best balance between model accuracy and interpretability.

Supplementary Table 2 Prediction accuracy of GPR models using different feature sets. Training and testing were repeated 100 times using different random states.

Test MAE*	Physicochemical feature	Extended connectivity fingerprint	Unique atom and coordination count (This work)
ASI	27.0 ± 9.4	19.4 ± 8.6	20.2 ± 6.9
ΔASI	20.9 ± 6.3	14.8 ± 7.5	16.7 ± 6.1
Q	16.7 ± 4.4	11.8 ± 4.8	12.2 ± 4.3
No. of features	66	2048	24

*Train:test = 80:20

Supplementary Table 3 Generated feature values for 28 additive combinations

Name	O_1	C_3 _inRing	O_2 _inRing	B[-1]_4 _inRing	F_1	Li[+1]	C_2 _inRing	B_3 _inRing	C_1	C_2	N_2 _inRing	N_3 _inRing	C_3	O[-1]_1	S_4 _inRing	P[-1]_4	Si_4	O_2	P_3	P[-1]_6 _inRing	P_4	C_4	S_4	N_2
LiDFOB_1+LDF_1	16	8	8	4	16	8	0	0	0	0	0	0	0	0	0	4	0	0	0	0	0	0	0	0
VC_1+LDF_1	12	4	8	0	8	4	8	0	0	0	0	0	0	0	0	4	0	0	0	0	0	0	0	0
PBE_1+LDF_1	8	4	8	0	8	4	32	4	0	0	0	0	0	0	0	4	0	0	0	0	0	0	0	0
tVCBO_0.25+LDF_1	8	0	3	0	8	4	5	3	3	3	1	0	0	0	0	4	0	0	0	0	0	0	0	0
LiDFOB_1+MS_1	16	16	16	4	24	8	4	0	0	0	0	0	0	0	0	0	0	0	0	4	0	0	0	0
VC_1+MS_1	12	12	16	0	16	4	12	0	0	0	0	0	0	0	0	0	0	0	0	4	0	0	0	0
PBE_1+MS_1	8	12	16	0	16	4	36	4	0	0	0	0	0	0	0	0	0	0	0	4	0	0	0	0
tVCBO_0.25+MS_1	8	8	11	0	16	4	9	3	3	3	1	0	0	0	0	0	0	0	0	4	0	0	0	0
LiDFOB_1+TMSPi_1	8	8	8	4	8	4	0	0	36	0	0	0	0	0	0	0	12	12	4	0	0	0	0	0
VC_1+TMSPi_1	4	4	8	0	0	0	8	0	36	0	0	0	0	0	0	0	12	12	4	0	0	0	0	0
PBE_1+TMSPi_1	0	4	8	0	0	0	32	4	36	0	0	0	0	0	0	0	12	12	4	0	0	0	0	0
tVCBO_0.25+TMSPi_1	0	0	3	0	0	0	5	3	39	3	1	0	0	0	0	0	12	12	4	0	0	0	0	0
tVCBO_0.25+None	0	0	3	0	0	0	5	3	3	3	1	0	0	0	0	0	0	0	0	0	0	0	0	0
LiDFOB_1+None	8	8	8	4	8	4	0	0	0	0	0	0	0	0	0	0	0	0	0	0	0	0	0	0
None+TMSPi_2	0	0	0	0	0	0	0	0	72	0	0	0	0	0	0	0	24	24	8	0	0	0	0	0
None+PTSI_0.5	6	4	0	0	0	0	8	0	2	2	0	0	0	0	0	0	0	0	0	0	0	0	2	2
None+DEPP_2	0	8	0	0	0	0	40	0	16	16	0	0	0	0	0	0	0	16	8	0	0	0	0	0
None+DPDMS_1	0	8	0	0	0	0	40	0	8	0	0	0	0	0	0	0	4	8	0	0	0	0	0	0
None+SA_3	24	24	12	0	0	0	24	0	0	0	0	0	0	0	0	0	0	0	0	0	0	0	0	0
None+TPP_10	40	120	0	0	0	0	600	0	0	0	0	0	0	0	0	0	0	120	0	0	40	0	0	0
PES_1+None	8	0	4	0	0	0	12	0	0	0	0	0	0	0	4	0	0	0	0	0	0	0	0	0
None+TMSPa_1	4	0	0	0	0	0	0	0	36	0	0	0	0	0	0	0	12	12	0	0	4	0	0	0
Li2C2O4_1+MS_1	16	8	8	0	16	12	4	0	0	0	0	0	8	8	0	0	0	0	0	4	0	0	0	0

LiBOB_1+LDF_1	24	16	16	4	8	8	0	0	0	0	0	0	0	0	0	4	0	0	0	0	0	0	0	0
ADM_1+LiOtC4F9_1	8	8	8	0	36	4	8	4	8	4	0	4	0	4	0	0	0	0	0	0	0	16	0	0
LiBOB_1+LiBFEP_1	20	16	16	4	24	8	0	0	0	8	0	0	0	4	0	0	0	8	0	0	4	8	0	0
LiDFOB_1+LiBFEP_1	12	8	8	4	32	8	0	0	0	8	0	0	0	4	0	0	0	8	0	0	4	8	0	0
ADM_1+TMSPi_1	8	8	8	0	0	0	8	4	44	4	0	4	0	0	0	0	12	12	4	0	0	0	0	0

Supplementary Note 5. Benchmark of ML algorithms

In addition to GPR, we evaluated the performance of several regression methods, including linear regression, gradient boosting, and random forest. To ensure robust results, we repeated the model training and testing process 100 times for each method, reporting the means and standard deviations of the test mean absolute error in Supplementary Table 5. Our findings indicate that GPR and Random Forest Regression provide comparable prediction accuracy, both outperforming gradient boosting (by a small margin) and linear regression (by a significant margin).

Supplementary Table 4 Prediction accuracy of different ML algorithms for ASI, ΔASI and Q. Training and testing were repeated 100 times using different random states.

Test MAE*	Linear Regression	Gradient Boosting	Random Forrest Regression	Gaussian Process Regression
ASI	39.9 ± 36.6	22.0 ± 9.0	20.4 ± 7.3	20.2 ± 6.9
ΔASI	20.7 ± 17.4	16.3 ± 6.3	15.6 ± 5.5	16.7 ± 6.1
Q	14.4 ± 10.3	12.9 ± 5.2	12.0 ± 4.1	12.2 ± 4.3

*Train:test = 80:20

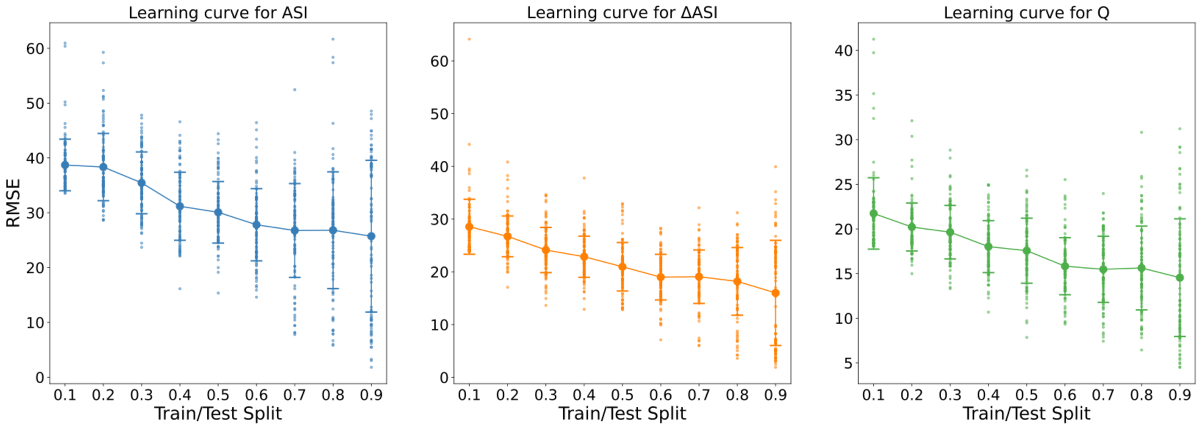

Supplementary Figure 4. Learning curves of Gaussian Process Regression (GPR) models. Prediction accuracy of GPR models evaluated at different train/test split ratios, averaged over 100 runs. Data are represented as mean values (solid circles) +/- one standard deviation (error bar). Source data are provided as a Source Data file.

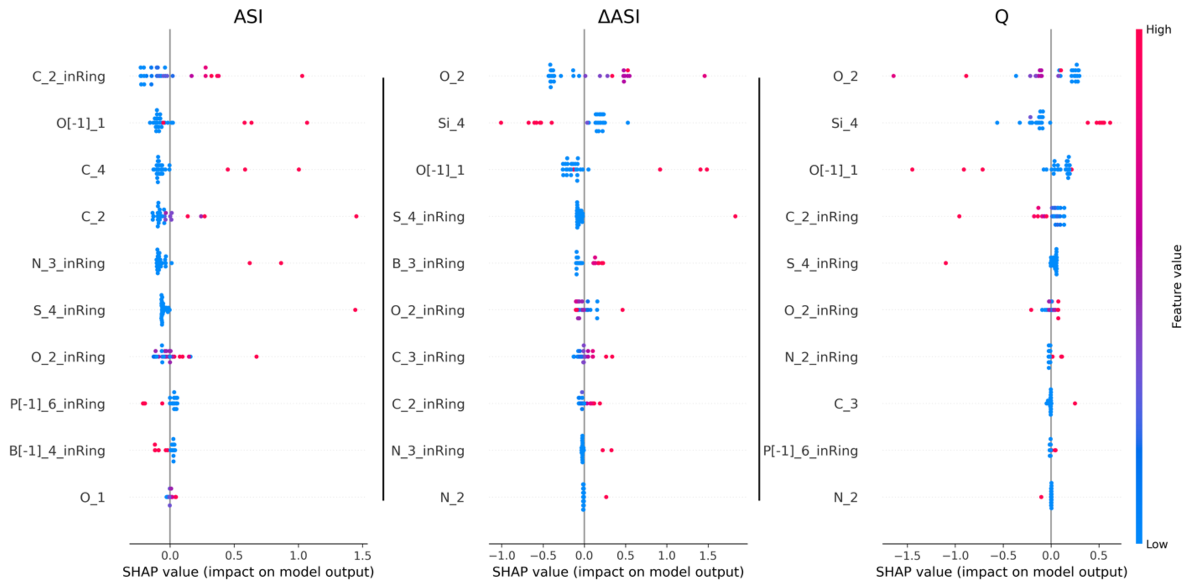

Supplementary Figure 5. SHAP analysis of model features with respect to the model predictions of ASI, ΔASI and Q

Supplementary Table 5 GPR-predicted values of impedance rise (ΔASI), final specific capacity (Q) and their corresponding stand deviation (SD).

**Additive**	**ΔASI**	**ΔASI _SD**	**Q**	**Q_SD**
LiDFOB_1+SA_1	18	20	88	9
LiBOB_1+TMSPi_1	19	20	88	9
LiBOB_1+MS_1	19	21	86	10
LiBOB_1+SA_1	19	20	86	10
VC_1+SA_1	20	20	86	9
LiBOB_1+LDF_1	19	21	84	10
LiDFOB_1+TMSPa_1	19	20	84	11
VC_1+TMSPa_1	19	19	83	11
LiBOB_1+TMSPa_1	19	20	82	12
PBE_1+TMSPa_1	30	21	78	12
PBE_1+SA_1	34	22	78	10
LiDFOB_1+DPDMS_1	34	22	77	10
LiBOB_1+DPDMS_1	34	23	76	11
VC_1+DPDMS_1	39	24	73	11
LiDFOB_1+MPS_1	49	39	71	17
LiBOB_1+MPS_1	49	39	70	17
VC_1+MPS_1	49	39	69	17
LiDFOB_1+DEPP_1	43	32	67	17
LiBOB_1+DEPP_1	43	33	66	17
LiDFOB_1+TPP_1	44	30	65	16
VC_1+DEPP_1	46	33	65	17

LiBOB_1+TPP_1	44	30	64	16
PBE_1+MPS_1	49	39	63	18
VC_1+TPP_1	46	33	62	17
PBE_1+DPDMS_1	47	34	62	15
LiDFOB_1+PTSI_1	49	39	62	18
VC_1+PTSI_1	49	39	62	18
LiBOB_1+PTSI_1	49	39	62	19
PBE_1+DEPP_1	52	36	59	19
PBE_1+PTSI_1	49	39	58	20
PES_1+TMSPi_1	63	28	57	13
PBE_1+TPP_1	49	38	56	20
PES_1+TMSPa_1	63	28	56	16
PES_1+MS_1	63	28	56	14
PES_1+LDF_1	63	28	56	14
PES_1+SA_1	63	28	56	13
PES_1+MPS_1	49	39	54	20
PES_1+DPDMS_1	55	37	54	18
PES_1+PTSI_1	49	39	53	22
PES_1+TPP_1	51	39	53	22
PES_1+DEPP_1	53	38	53	22
tVCBO_1+LDF_1	53	37	52	22
tVCBO_1+TMSPi_1	54	37	52	21
tVCBO_1+MS_1	54	37	52	22
tVCBO_1+TMSPa_1	54	37	52	22
tVCBO_1+SA_1	55	37	52	21
LiDFOB_1+DDD_1	49	39	51	23
LiDFOB_1+LiBFEP_1	49	39	51	23
LiDFOB_1+BA_1	49	39	51	23
LiDFOB_1+LiOtC4F9_1	49	39	51	23
VC_1+DDD_1	49	39	51	23
VC_1+LiBFEP_1	49	39	51	23
VC_1+BA_1	49	39	51	23
VC_1+LiOtC4F9_1	49	39	51	23
PBE_1+DDD_1	49	39	51	23
PBE_1+LiBFEP_1	49	39	51	23
PBE_1+BA_1	49	39	51	23
PBE_1+LiOtC4F9_1	49	39	51	23
tVCBO_1+DDD_1	49	39	51	23
tVCBO_1+LiBFEP_1	49	39	51	23
tVCBO_1+BA_1	49	39	51	23
tVCBO_1+MPS_1	49	39	51	22
tVCBO_1+LiOtC4F9_1	49	39	51	23

VTA_1+LDF_1	49	39	51	23
VTA_1+TMSPi_1	49	39	51	23
VTA_1+MS_1	49	39	51	23
VTA_1+DDD_1	49	39	51	23
VTA_1+LiBFEP_1	49	39	51	23
VTA_1+BA_1	49	39	51	23
VTA_1+PTSI_1	49	39	51	23
VTA_1+MPS_1	49	39	51	23
VTA_1+LiOtC4F9_1	49	39	51	23
VTA_1+DEPP_1	49	39	51	23
VTA_1+DPDMS_1	49	39	51	23
VTA_1+SA_1	49	39	51	23
VTA_1+TPP_1	49	39	51	23
VTA_1+TMSPa_1	49	39	51	23
Vim_1+LDF_1	49	39	51	23
Vim_1+TMSPi_1	49	39	51	23
Vim_1+MS_1	49	39	51	23
Vim_1+DDD_1	49	39	51	23
Vim_1+LiBFEP_1	49	39	51	23
Vim_1+BA_1	49	39	51	23
Vim_1+PTSI_1	49	39	51	23
Vim_1+MPS_1	49	39	51	23
Vim_1+LiOtC4F9_1	49	39	51	23
Vim_1+DEPP_1	49	39	51	23
Vim_1+DPDMS_1	49	39	51	23
Vim_1+SA_1	49	39	51	23
Vim_1+TPP_1	49	39	51	23
Vim_1+TMSPa_1	49	39	51	23
LiBOB_1+DDD_1	49	39	51	23
LiBOB_1+LiBFEP_1	49	39	51	23
LiBOB_1+BA_1	49	39	51	23
LiBOB_1+LiOtC4F9_1	49	39	51	23
Li2C2O4_1+LDF_1	49	39	51	23
Li2C2O4_1+TMSPi_1	49	39	51	23
Li2C2O4_1+MS_1	49	39	51	23
Li2C2O4_1+DDD_1	49	39	51	23
Li2C2O4_1+LiBFEP_1	49	39	51	23
Li2C2O4_1+BA_1	49	39	51	23
Li2C2O4_1+PTSI_1	49	39	51	23
Li2C2O4_1+MPS_1	49	39	51	23
Li2C2O4_1+LiOtC4F9_1	49	39	51	23
Li2C2O4_1+DEPP_1	49	39	51	23

Li2C2O4_1+DPDMS_1	49	39	51	23
Li2C2O4_1+SA_1	49	39	51	23
Li2C2O4_1+TPP_1	49	39	51	23
Li2C2O4_1+TMSPa_1	49	39	51	23
ADM_1+LDF_1	49	39	51	23
ADM_1+TMSPi_1	49	39	51	23
ADM_1+MS_1	49	39	51	23
ADM_1+DDD_1	49	39	51	23
ADM_1+LiBFEP_1	49	39	51	23
ADM_1+BA_1	49	39	51	23
ADM_1+PTSI_1	49	39	51	23
ADM_1+MPS_1	49	39	51	23
ADM_1+LiOtC4F9_1	49	39	51	23
ADM_1+DEPP_1	49	39	51	23
ADM_1+DPDMS_1	49	39	51	23
ADM_1+SA_1	49	39	51	23
ADM_1+TPP_1	49	39	51	23
ADM_1+TMSPa_1	49	39	51	23
PES_1+DDD_1	49	39	51	23
PES_1+LiBFEP_1	49	39	51	23
PES_1+BA_1	49	39	51	23
PES_1+LiOtC4F9_1	49	39	51	23
tVCBO_1+PTSI_1	49	39	50	23
tVCBO_1+TPP_1	52	39	50	23
tVCBO_1+DPDMS_1	55	38	50	22
tVCBO_1+DEPP_1	57	37	47	22

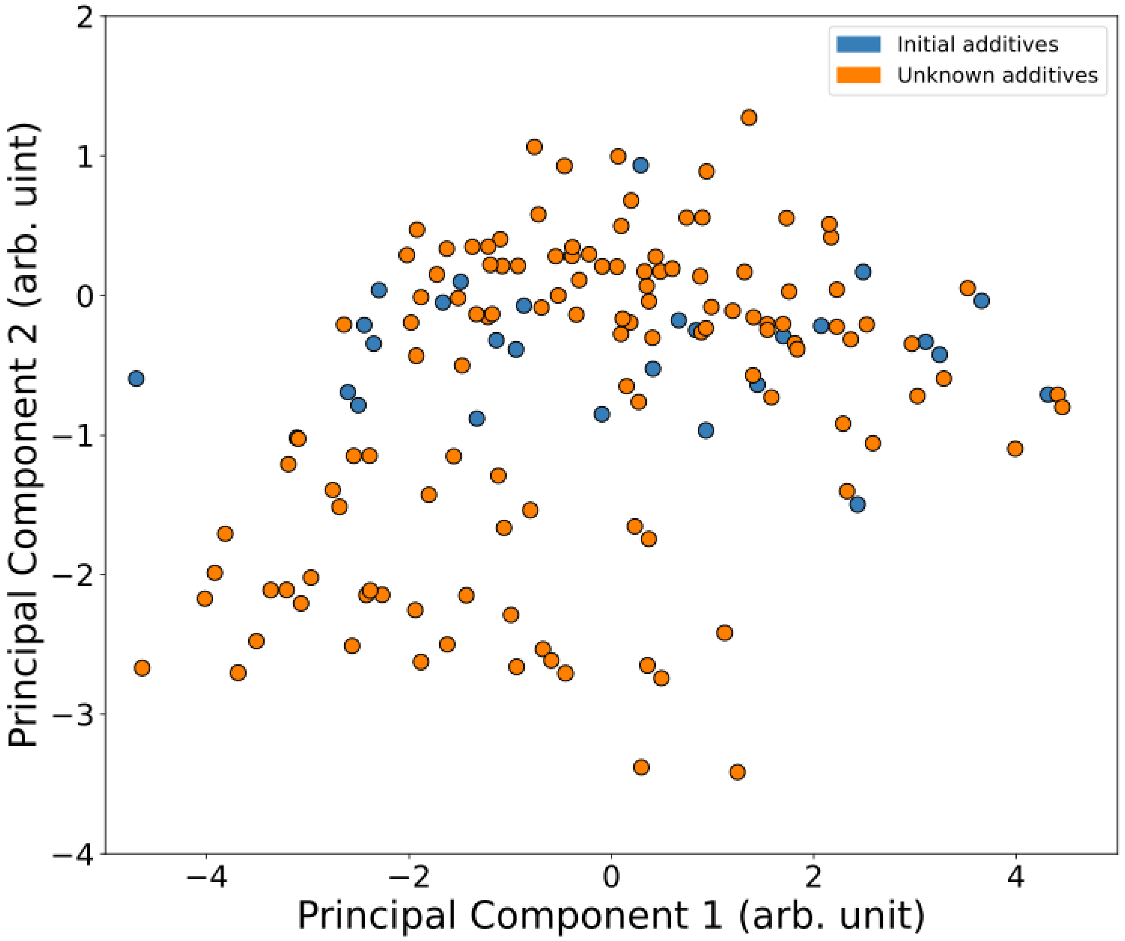

Supplementary Figure 6. Principal component analysis. Graphical illustration of the feature space of all considered additives projected onto principal component 1 and 2. Blue and orange circles represent initial and unknown additives.

Supplementary Note 6. Justification of the use of machine learning (ML) for 125 unknown additives

While conducting all 125 experiments would yield most accurate results, it is important to consider that each additive requires triplicate cells, and each cell must be tested using a dedicated MACCOR channel. Besides being time-consuming, the need for 375 (125 × 3) MACCOR channels with temperature control would drive the equipment cost to over $500,000, without reducing the testing duration.

Supplementary Note 7. Selection of predicted additive candidates via Exploitation and Bayesian optimization

In addition to exploitation, which follows the local optima (of predicted ∆ASI and Q),we also incorporated Bayesian optimization to suggest potential additive systems for experimental validation. Bayesian optimization is a method that utilizes an acquisition function to calculate the fitness rank of unknown candidates based on the output of a surrogate model (e.g., Gaussian Process Regression). Different acquisition functions may be employed in Bayesian optimization including upper confidence bound, probability of improvement, and expected improvement. Regardless, these functions allow Bayesian optimization to strike a good balance between exploration and exploitation.

Supplementary Table 6 Top additive systems predicted using Exploitation and Bayesian optimization.

Fitness rank	Exploitation	Bayesian optimization		
		**Upper Confidence Bound**	**Probability of Improvement**	**Expected Improvement**
1	LiDFOB_1+SA_1	LiDFOB_1+SA_1	LiDFOB_1+SA_1	LiDFOB_1+SA_1
2	LiBOB_1+TMSPi_1	LiBOB_1+TMSPi_1	LiBOB_1+TMSPi_1	LiBOB_1+TMSPi_1
3	LiBOB_1+MS_1	LiBOB_1+MS_1	LiBOB_1+MS_1	LiBOB_1+MS_1
4	LiBOB_1+SA_1	LiBOB_1+SA_1	LiBOB_1+SA_1	LiBOB_1+SA_1
5	VC_1+SA_1	VC_1+SA_1	VC_1+SA_1	LiDFOB_1+TMSPa_1
6	LiBOB_1+LDF_1	LiDFOB_1+TMSPa_1	LiDFOB_1+TMSPa_1	LiBOB_1+TMSPa_1

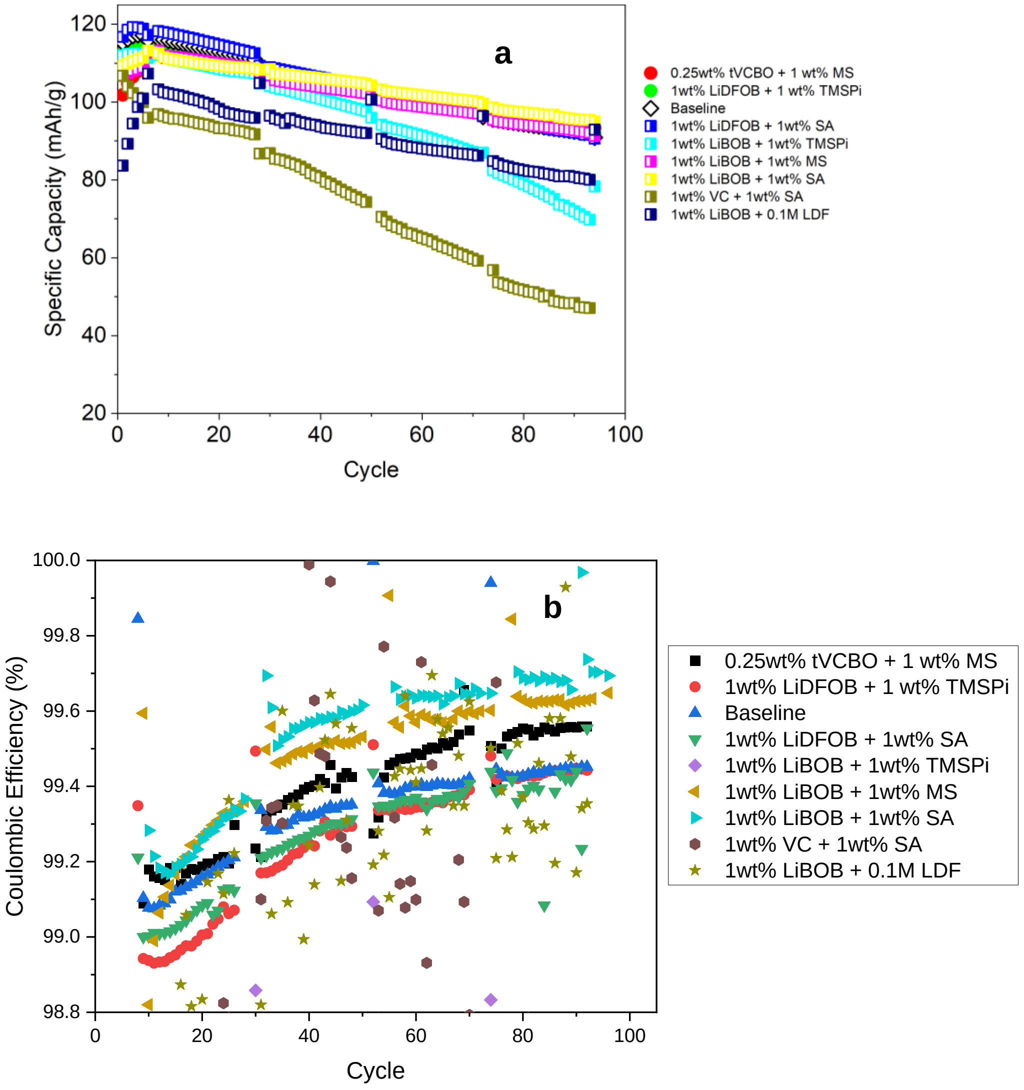

Supplementary Figure 7. Electrochemistry of Gr||LNMO using ML-predicted additives. (a) Capacity retention and (b) Coulombic efficiencies of Gr||LNMO using ML-predicted additives. The data represents the average of three cells for each formulation, and the coin cells were cycled at 30 ^{o}C with the protocol mentioned in Supplementary Note 1.

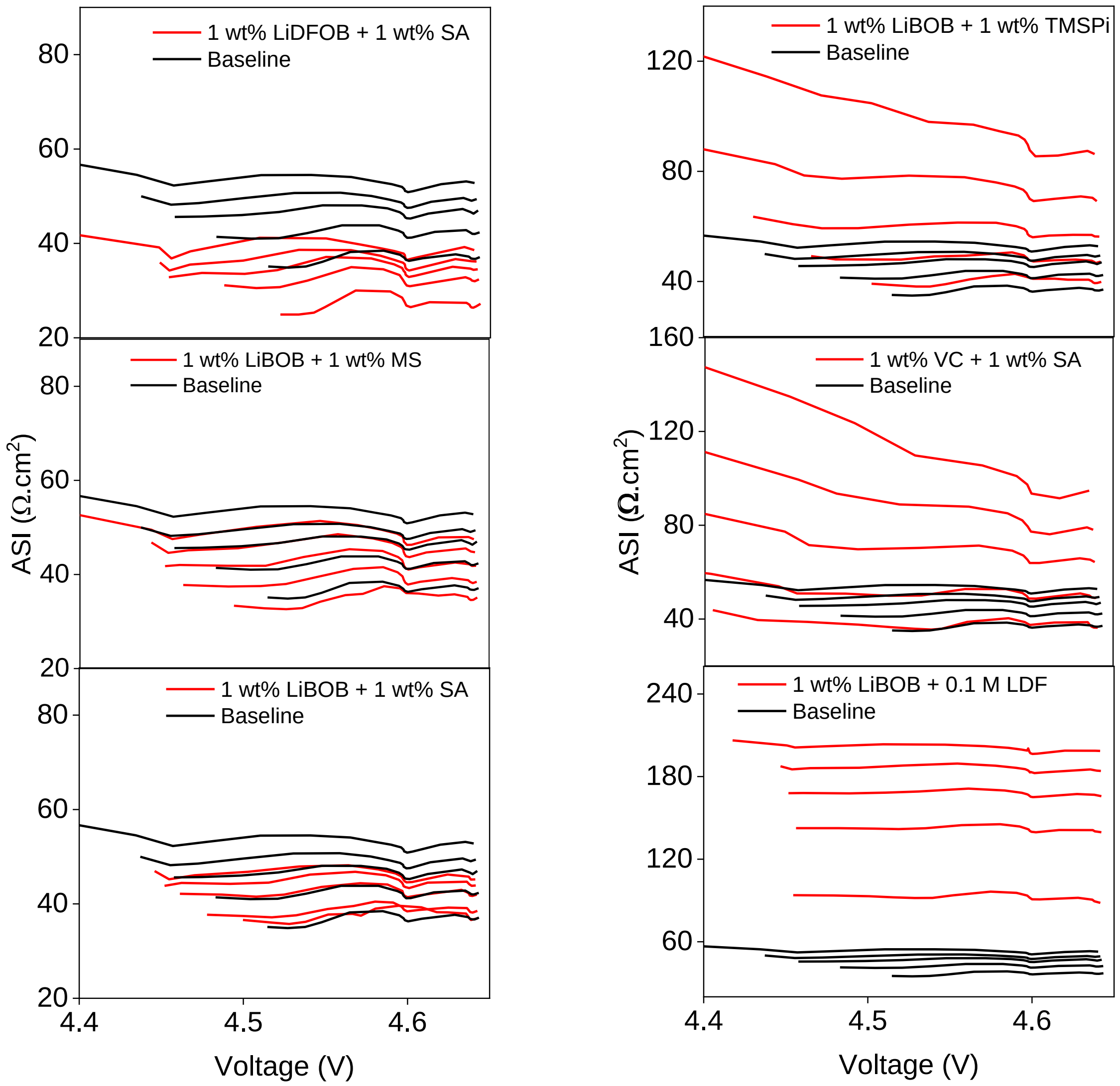

Supplementary Figure 8. The HPPC (Hybrid Pulse Power Characterization) data of the ML-predicted additives is presented here. Three additives demonstrate superior performance compared to the baseline electrolyte of 1.0 M LiPF6 in EC/EMC (1/9): 1 wt% LiDFOB + 1 wt% SA, 1 wt% LiBOB + 1 wt% MS, and 1 wt% LiBOB + 1 wt% SA.

Supplementary Note 8. Capacity Loss Mechanisms demonstrated by various methods

8.1 Diagnostic Cycling of the Harvested Cells

A specific protocol was implemented to investigate the amount of lithium inventory trapped in the anode/negative electrode. In this protocol, the cell was disassembled when it reached the discharged state, with all cyclable lithium inventory remaining in the positive electrode. Subsequently, both the positive and negative electrodes were paired with fresh lithium metal and fresh lithium electrolyte (1.0 M $LiPF_6$ in EC/EMC, 1/9 v/v). Note that neither of the discs were washed during the disassembly process in order to preserve the primary morphologies and compositions of the potential Solid Electrolyte Interface (SEI) and Cathode Electrolyte Interphase (CEI), as well as the grain boundaries. This preservation helps in analyzing the properties and behavior of these interfaces.

The protocol for the cathode/positive electrode involved an initial discharge step at a rate of C/100 (hundredth of the capacity of the cell) followed by one charge and one discharge cycle at the same rate.

By following this protocol, we were able to study the amount of lithium inventory that remained trapped in the negative electrode and investigate the behavior of these positive electrode under controlled conditions. Note also only the cells with the best performers are subjected to this procedure, namely, 0.25wt% tVCBO + 1wt% MS, 1wt% LiDFOB +1wt% TMSPi, 1wt% LiBOB + 1wt% MS, and 1wt% LiBOB + 1wt% SA.

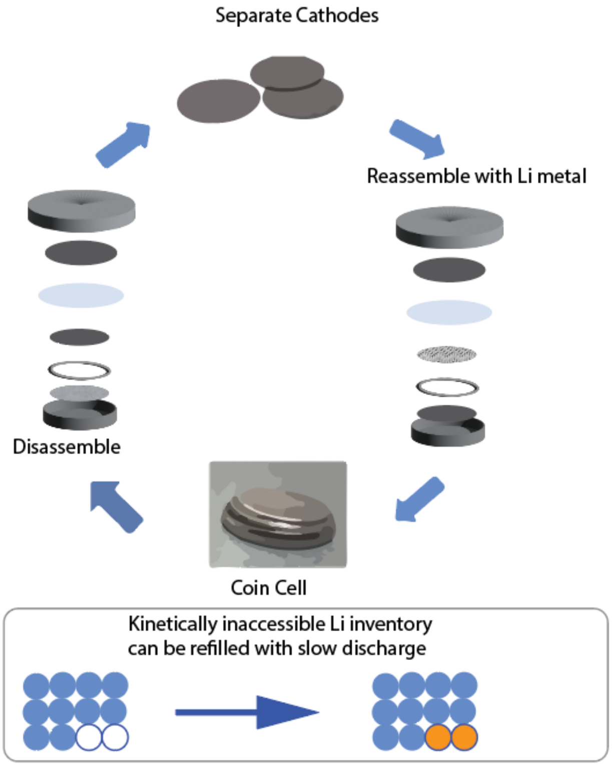

Supplementary Figure 9. Diagnosis through disassembly and reassembly of the harvested electrode. Schematic illustration to demonstrate the inaccessible site of Li (orange dots) that can be refilled at a slow rate.

The initial results of the kinetically accessible Li inventory obtained by electrochemical cycling are shown in Supplementary Table 7. From Supplementary Table 7, the baseline solution shows significant loss of inventory in the positive electrodes. The consumption of Li inventory during the SEI formation was most in baseline, and much less in the electrolyte containing 0.25wt% tVCBO + 1wt% MS and 1wt% LiDFOB +1wt% TMSPi. The table further confirms that the difference observed is mainly due to the loss of inventory in the positive electrode that is exacerbated by transition metal (TM) dissolution.

Supplementary Table 7 The Li inventory content calculated from the first charge, first discharge and final charge capacity.

Electrolytes	Kinetically inaccessible Li inventory (mAh g^{-1})[a]	First Charge capacity (mAh g^{-1})	First Discharge capacity (mAh g^{-1})	Irreversible capacity for 1st cycle (SEI) (mAh g^{-1})[b]	Final discharge capacity (mAh g^{-1})	Loss of inventory in positive electrode (mAh g^{-1})[c]
**Baseline**	13.2	133.8	122.0	11.7	74.8	47.3
**0.25wt% tVCBO+ 1wt% MS**	2.5	144.5	133.1	11.3	90.7	42.4
**1wt% LiDFOB + 1wt% TMSPi**	0.8	146.2	132.5	13.6	91.3	41.2
**1wt% LiBOB + 1wt% MS**	0.9	146.1	136.0	10.1	97.8	38.2
**1wt% LiBOB + 1wt% SA**	2.1	144.9	135.1	9.7	97.6	37.6

a) The kinetically inaccessible Li inventory was calculated by subtracting first charge from the theoretical capacity of 147 mAh g^{-1};
b) Irreversible capacity for 1st cycle was calculated by subtracting first discharge capacity from first charge capacity.
c) Loss of inventory was calculated by subtracting final discharge capacity from first charge capacity.

Using the approach outlined in Supplementary Fig. 9 , we also conducted experiments to gain insights into lithium inventory loss by performing slow cycling tests (C/100) on reassembled cells containing aged positive electrodes and a fresh lithium chip. As depicted in Supplementary Fig. 12, the results demonstrate that the aged positive electrode that is coupled with fresh Li (blue) exhibited high specific capacities, surpassing the specific capacities of the last slow C/25 RPT cycles. This observation indicates that the degradation mechanism is primarily attributed to lithium inventory loss rather than the loss of positive electrode active materials. The performance of other cells utilizing additives, such as 1 wt% LiDFOB + 1 wt% TMSPi and 1 wt% LiBOB + 1 wt% SA, is shown in Supplementary Fig. 10.

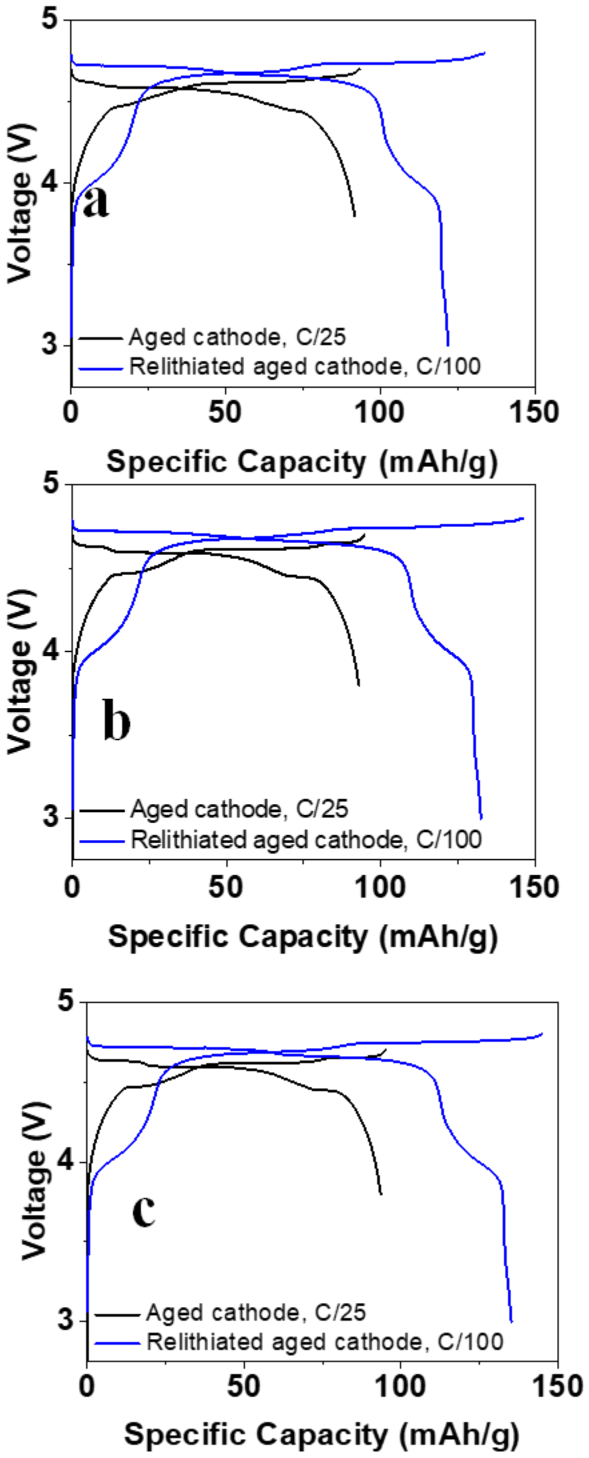

Supplementary Figure 10. Electrochemistry of reassembled coin cells using the harvested positive electrodes. Compared cycling performance of aged positive electrode at 94th cycle (black) vs aged positive electrode reassembled with a fresh Li metal negative electrode (blue). The cell was cycled with a) Baseline b) 1wt% LiDFOB + 1wt% TMSPi, and c) 1wt% LiBOB + 1wt% SA.

8.2 ^{1}H Nuclear Magnetic Resonance

^{1}H spectroscopy serves as a valuable method for detecting side products such as transesterification or hydrolysis that are generated in the electrolyte during cycling. Transesterification involves the interchange of carbonate esters, wherein the side products of diethyl carbonate (DEC) and dimethylcarbonate (DMC) indicate the self-exchange between two EMC molecules. These techniques provide important insights into the presence and nature of side reactions occurring during the cycling process. Clearly, the presence of additives inhibited the formation of transesterification products (Supplementary Fig. 11).

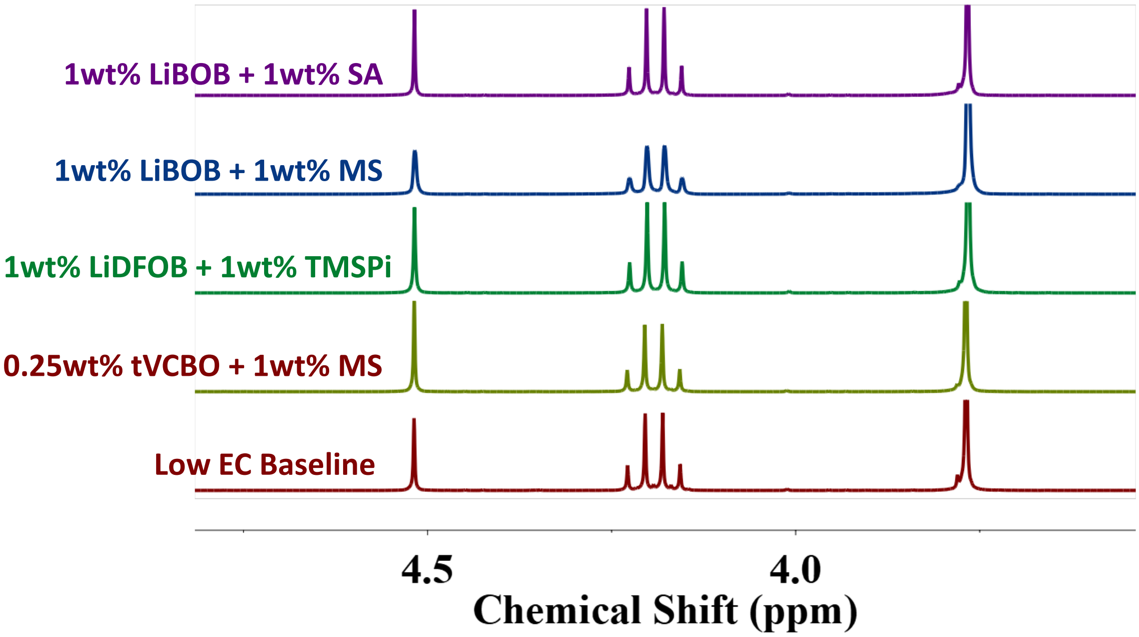

Supplementary Figure 11. ^{1}H NMR spectra of the aged electrolytes in $CDCl_3$. The results indicate the baseline (Low EC) after cycling shows formation of transesterification products , while addition of additives (1 wt% LiBOB + 1 wt% SA, 1 wt% LiBOB + 1 wt% MS, 1 wt% LiDFOB + 1 wt% TMSPi, and 0.25 wt% tVCBO + 1 wt% MS) help the carbonate solvents to avoid transesterification products.

8.3 X-ray Photoelectron Spectroscopy (XPS)

XPS analysis provides valuable information on the chemical composition and bonding at the outermost layer of the cathode/positive electrode laminate, including the formation of the SEI and cathode-electrolyte interface (CEI). Our previous XPS study revealed a correlation between the content of oxyfluorophosphate ($LiPO_xF_y$) on the positive electrode surface and the beneficial effect on impedance reduction. Here, the CEI contents of aged positive electrodes were examined by XPS from the best performing additives (0.25 wt% tVCBO + 1 wt% MS and 1 wt% LiDFOB + 1 wt% TMSPi) and compared to that of the baseline. The XPS spectra (Supplementary Fig. 12) clearly demonstrate that the presence of these additives promotes the formation of oxyfluorophosphate (indicated by a peak at 135 eV in the *P2p* region) and LiF (indicated by a peak at 685 eV in the *F1s* region), leading to improved CEI quality and a significant reduction in impedance.

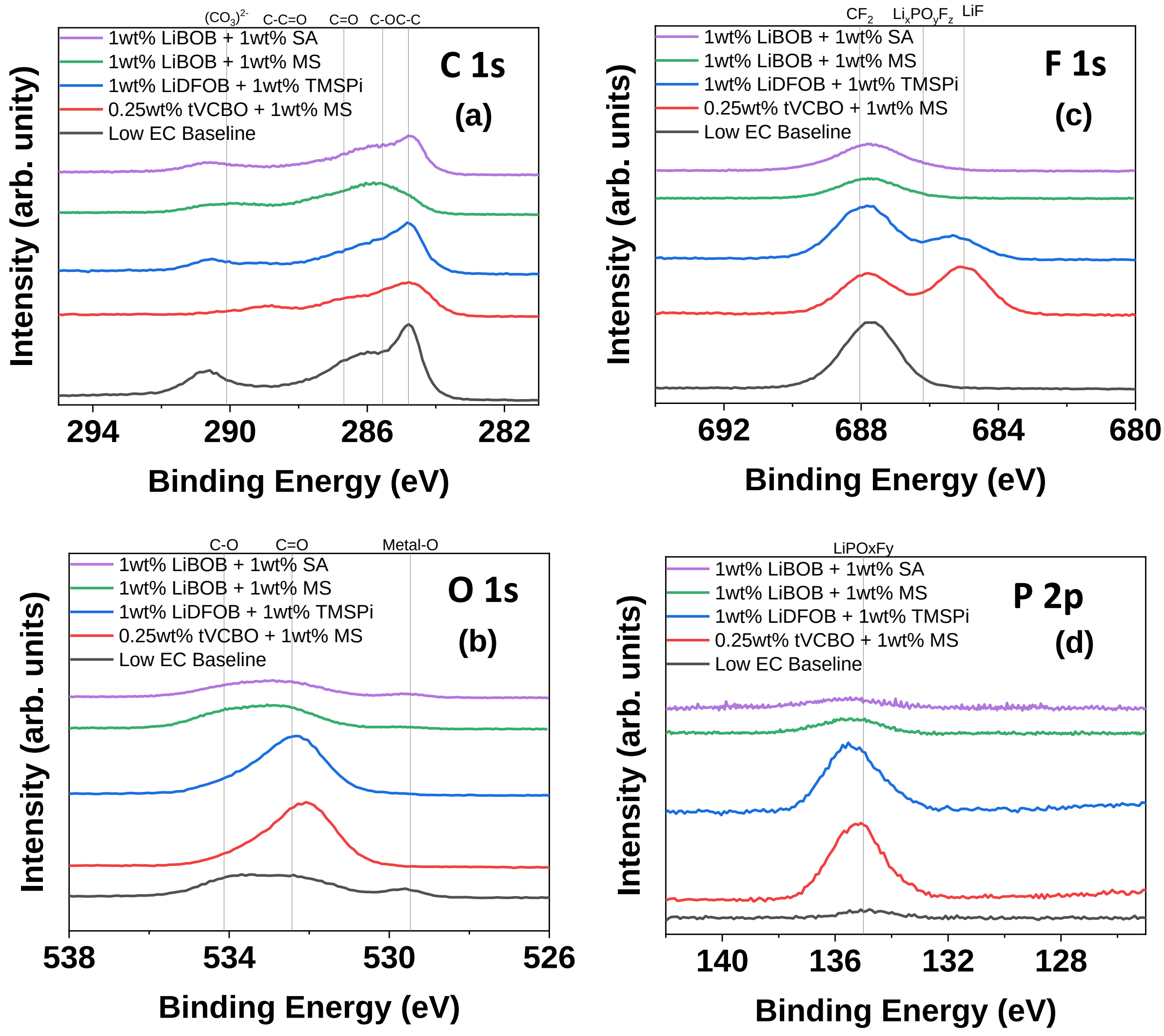

Supplementary Figure 12. X-ray photoelectron spectroscopy (XPS) of the cycled positive electrodes. The C 1s, O 1s, F 1s and P 2p regions of XPS spectra of cycled positive electrodes: (a) C 1s region; (b) O 1s region; (c) F 1s region; (d) P 2p region. The legend shows the baseline electrolytes 1 (black), 1 wt% LiBOB + 1 wt% SA (purple), 1 wt% LiBOB + 1 wt% MS (green), 1 wt% LiDFOB + 1 wt% TMSPi (blue), 0.25 wt% tVCBO + 1 wt% MS (red).

8.4 Inductively Coupled Plasma Mass Spectrometry

A direct comparison of transition metal dissolution from the positive electrode and its subsequent migration to the negative electrode side can be observed through the ICP-MS data obtained from the negative electrodes in the aged cell. Supplementary Fig. 13 demonstrates that the implementation of additives results in enhanced protection of positive electrode surfaces, as evidenced by the reduced quantities of Mn and Ni detected in the negative electrodes.

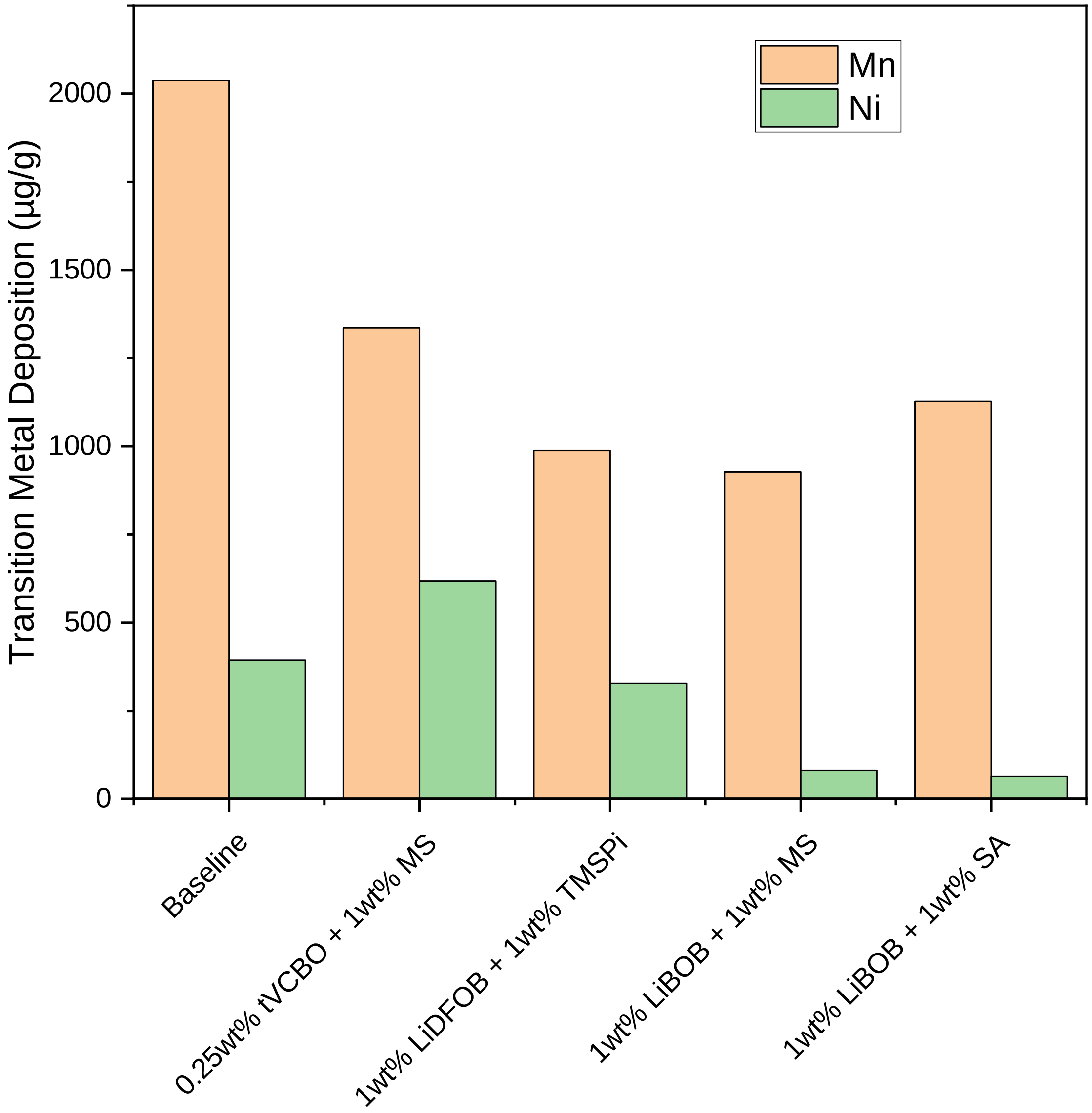

Supplementary Figure 13. Elemental analysis of electrolytes through ICP-MS. ICP-MS spectra of electrolytes extracted from cycled cells.

8.5 Scanning Electron Microscopy and Energy-dispersive X-ray spectroscopy (EDX) analysis

Another characterization technique is the SEM and EDX mapping analysis of the cycled negative electrodes. As shown in Supplementary Fig. 14-18, the SEM images also indicate a more uniformly formed SEI when additives are present, resulting in reduced loss of lithium inventory.[16] The concept of the high surface areal lithium (HSAL) refers to the formation of lithium dendrites during the cycling, a phenomenon observed in the Gr||TMO full cells. By using SEM to study the morphology of the cycled graphite negative electrodes, a correlation was established between the superior performance of certain additives and the inhibition of HSAL, leading to formation of a more uniform lithiated graphites. Although in the Gr||LNMO cells no typical rollover behavior is observed, yet significant incoherence and significant variations exist among triplicate cells. As shown in Supplementary Fig. 14-18, the aged graphite negative electrode displays areas with different shades of SEI under SEI, indicating the composition differences after cycling.

As shown in Supplementary Fig. 14, the SEI formed on graphite in the cells using baseline is not uniform, with regions rich in O, F, and Mn while poor in C exist. While in the presence of the best-performing additives (four formulations of 0.25wt% tVCBO + 1wt% MS, 1wt% LiDFOB + 1wt% TMSPi, 1wt% LiBOB + 1wt% MS, and 1wt% LiBOB + 1wt% SA), the graphite negative electrode exhibits uniform and compact SEI (Supplementary Fig. 15-18). Furthermore, the cycled negative electrode with 0.25wt% tVCBO + 1wt% MS (Supplementary Fig. 15) shows accumulated island-like TM in the SEM images, agreeing with the previous report on the ability of additives to aggregate

the TM dissolution and mitigate their overall effect on reducing lithium inventory loss. Particularly in the TMSPi case, visible Si deposits on the graphite suggest that the aggregates originate from the decomposition from the 1wt% LiDFOB + 1wt% TMSPi additive (Supplementary Fig. 16).

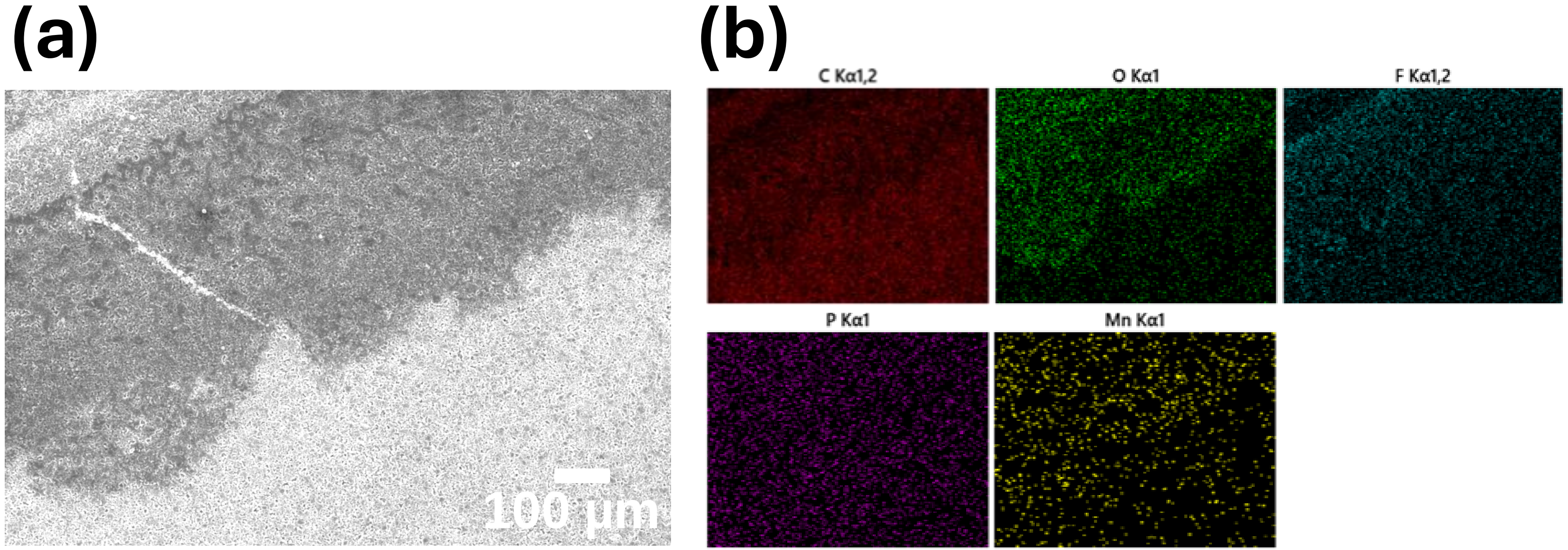

Supplementary Figure 14. Details of SEI on graphite negative electrode. SEM image (a) and the EDX mapping (b) of the cycled Gr negative electrode using the baseline. The image shows in cycled Gr 1) there are areas with different shades of SEI under SEI, indicating the composition differences 2) after cycling there is no cluster of Mn rich particles.

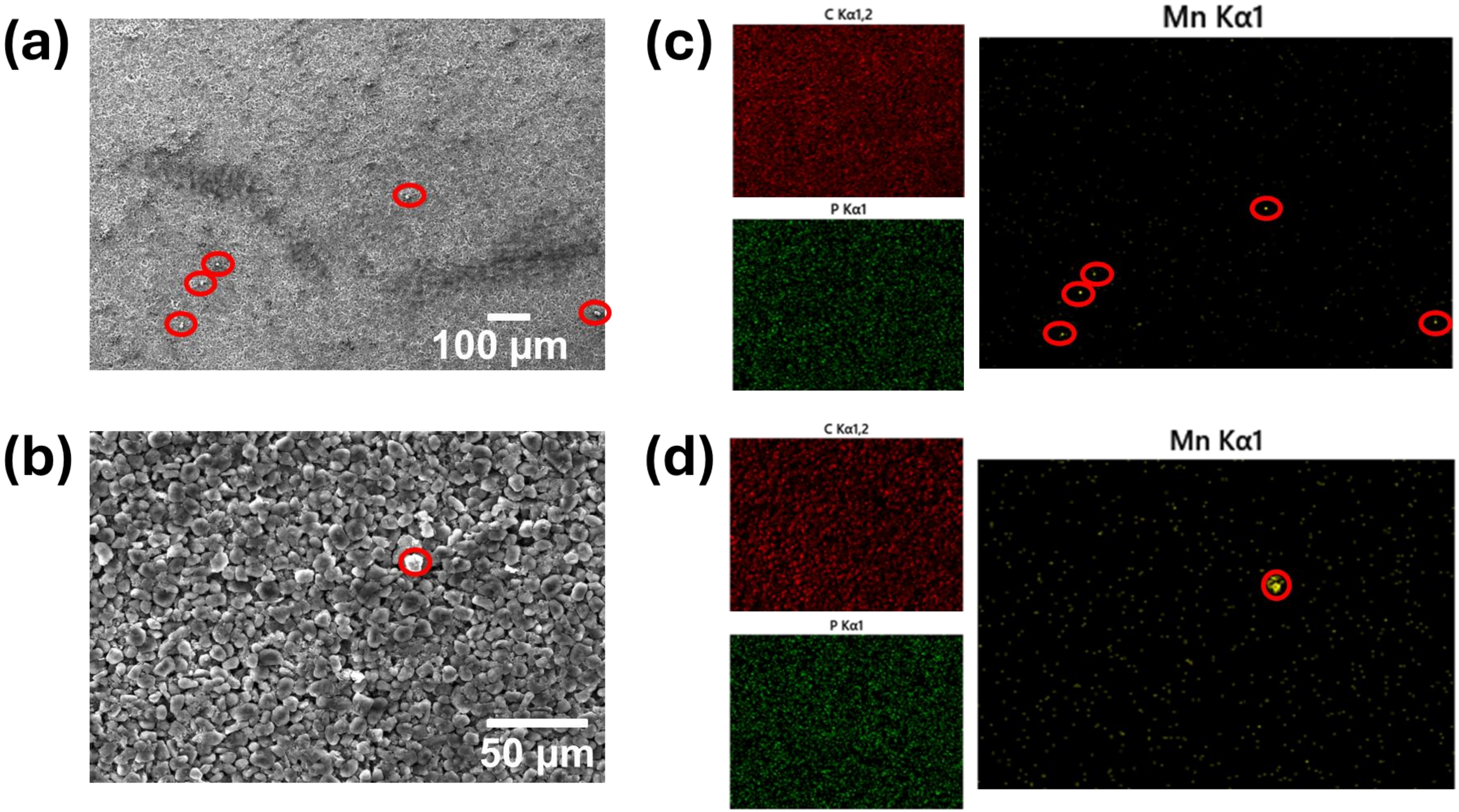

Supplementary Figure 15. Details of SEI on graphite negative electrode. SEM images (a, b) and the EDX mappings (c, d) of the cycled Gr negative electrode using the electrolyte with 0.25wt% tVCBO + 1wt% MS. cluster of Mn rich particles are presented in red cycles.

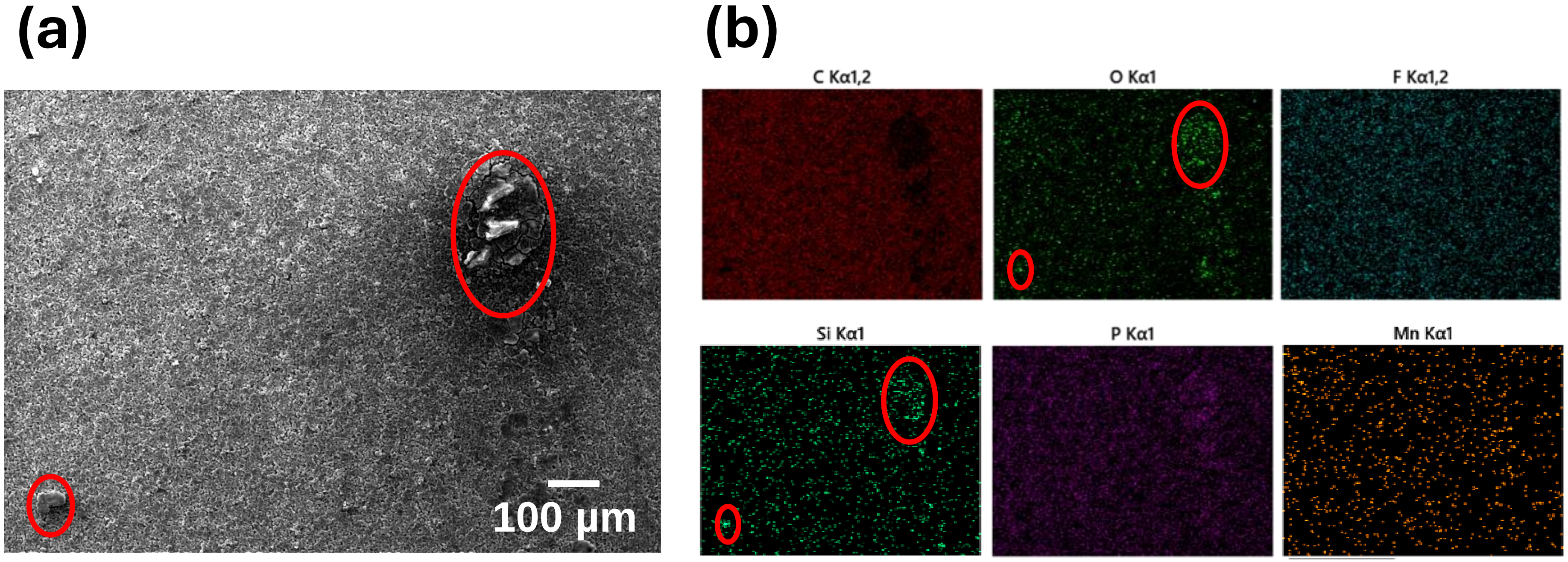

Supplementary Figure 16. Details of SEI on graphite negative electrode. SEM image (a) and the EDX mapping (b) of the cycled Gr negative electrode using the electrolyte with 1wt% LiDFOB + 1wt% TMSPi. Si and O rich particle depositions are presented in red cycles.

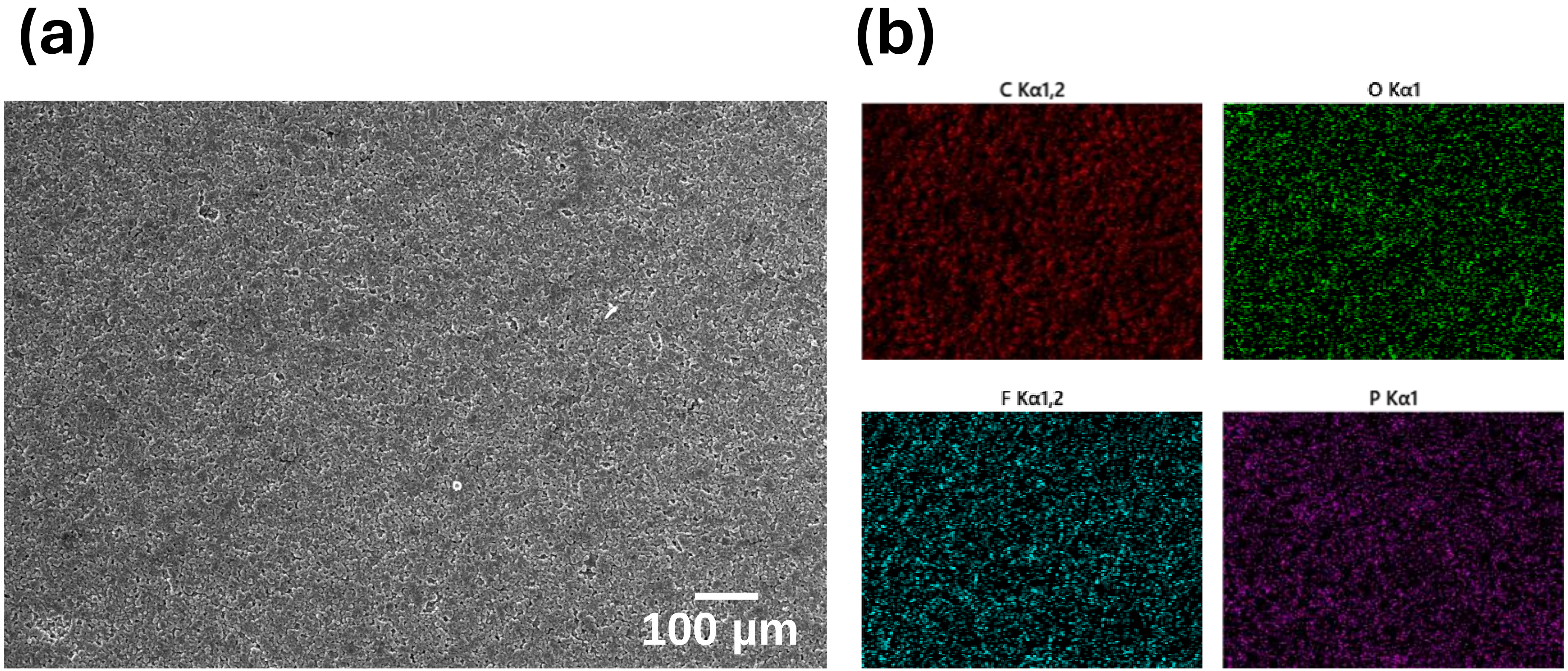

Supplementary Figure 17. Details of SEI on graphite negative electrode. SEM image (a) and the EDX mapping (b) of the cycled Gr negative electrode using the electrolyte with 1wt% LiBOB + 1wt% MS. The image shows in cycled Gr using the electrolyte with 1wt% LiBOB + 1wt% MS, the SEI is uniform.

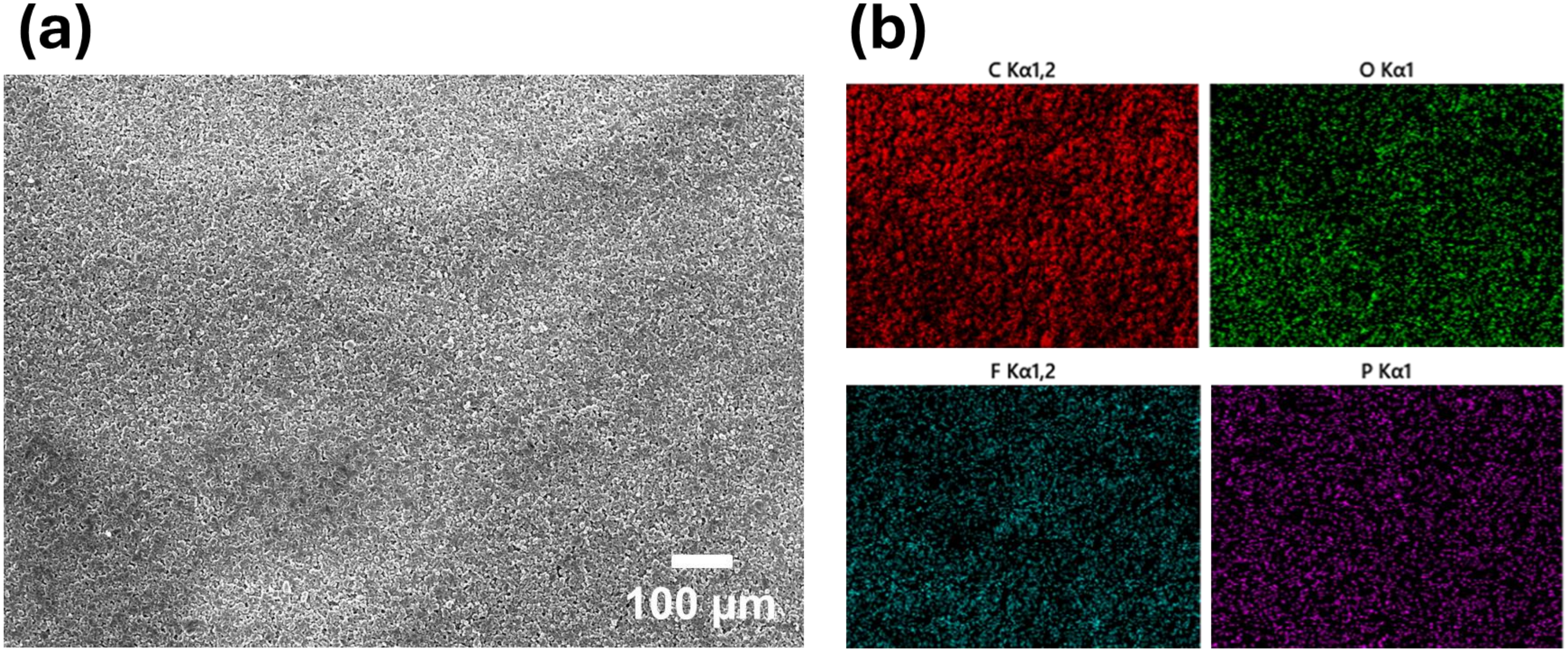

Supplementary Figure 18. Details of SEI on graphite negative electrode. *SEM image (a) and the EDX mapping (b) of the cycled Gr* negative electrode *using the electrolyte with 1wt% LiBOB + 1wt% SA. The image shows in cycled Gr using the electrolyte with 1wt% LiBOB + 1wt% SA, the SEI is uniform.*

8.6 Online Electrochemical Mass Spectrometry (OEMS)

To understand the fading mechanism, diagnostic experiments were carried out on OEMS to monitor H_2 evolution through different stages of cycling. Through an in-house set up of OEMS, the effective formation of H_2 gas evolution is clearly observed at the high state of charge (SOC), particularly during the high voltage hold period. This observation aligns with the previous report indicating a close relationship between H_2 content generation and graphite lithiation and LNMO delithiation. Our OEMS system utilized a modified version of capillary based online electrochemical mass spectrometer that does not disturb the pressure inside the gas line. Therefore, the pressure change of the OEMS directly reflects the amount of the gas generated, and the system allows complete evacuation and flushing of the system with He, as well as the real-time calibration for gas quantification.[17] By comparing the H2 evolution of the full cells during the first two galvanostatic cycles using additives (0.25 wt% tVCBO + 1 wt% MS, 1 wt% LiBOB + 1 wt% MS, 1 wt% LiBOB + 1 wt% SA) and the baseline, it is demonstrated that interfacial reactions on the negative and positive electrodes closely relate to H_2 evolution (Supplementary Fig. 14). In the first cycle, the first significant H_2 evolution occurs during the high voltage potentiostatic hold at ~ 23 hours (marked as ❶), which is attributed to the H_2O reduction during the high voltage hold. More interestingly is the second H2 evolution, which is found at the end of the discharge step (marked as ❷). This step, according to Janek et al, is caused by the breakdown of the SEI and consequently reduction of solvent during the rebuilding of the SEI. In the 0.25 wt% tVCBO + 1 wt% MS additive case, however, the ❷ H_2

evolution is much suppressed, indicating a much robust SEI with the presence of such additives.

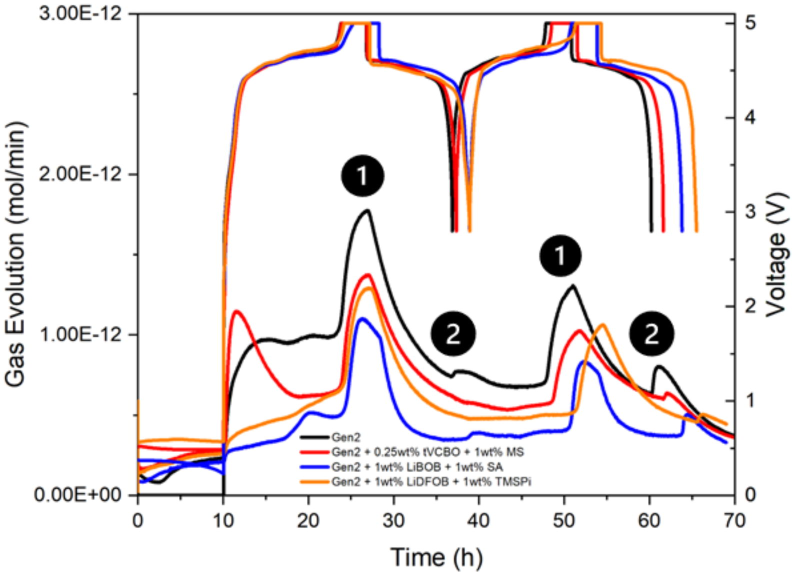

Supplementary Figure 19. Demonstration of breakdown of the SEI and consequently reduction during the rebuilding of the SEI. OEMS measurement of H_2 gas evolution during the first two galvanostatic cycles with Gr||LNMO cells. H_2 evolution is much suppressed in presence of additives, indicating a much robust SEI with the presence of such additives.

Supplementary References

1. Long, B. R.; Rinaldo, S. G.; Gallagher, K. G.; Dees, D. W.; Trask, S. E.; Polzin, B. J.; Jansen, A. N.; Abraham, D. P.; Bloom, I.; Bareño, J.; Croy, J. R., Enabling High-Energy, High-Voltage Lithium-Ion Cells: Standardization of Coin-Cell Assembly, Electrochemical Testing, and Evaluation of Full Cells. *Journal of The Electrochemical Society* **2016,** *163* (14), A2999.
2. Wang, B.; Gim, J.; Son, S.-B.; Shkrob, I. A.; Abraham, D. P.; Trask, S. E.; Qin, Y.; Kahvecioglu, O.; Jansen, A. N.; Liao, C., Electrolyte Study for High-Nickel LiNi0.9Mn0.05Co0.05O2 Cathodes. *Journal of The Electrochemical Society* **2023,** *170* (2), 020505.
3. Bloom, I.; Cole, B. W.; Sohn, J. J.; Jones, S. A.; Polzin, E. G.; Battaglia, V. S.; Henriksen, G. L.; Motloch, C.; Richardson, R.; Unkelhaeuser, T.; Ingersoll, D.; Case, H. L., An accelerated calendar and cycle life study of Li-ion cells. *Journal of Power Sources* **2001,** *101* (2), 238-247.
4. Wang, B.; Son, S.-B.; Badami, P.; Trask, S. E.; Abraham, D.; Qin, Y.; Yang, Z.; Wu, X.; Jansen, A.; Liao, C. Understanding and Mitigating the Dissolution and Delamination Issues Encountered with High-Voltage LiNi0.5Mn1.5O4 *Batteries* [Online], 2023.
5. Bizot, C.; Blin, M.-A.; Guichard, P.; Soudan, P.; Gaubicher, J.; Poizot, P., Aluminum current collector for high voltage Li-ion battery. Part II: Benefit of the En' Safe® primed current collector technology. *Electrochemistry Communications* **2021,** *126*, 107008.
6. Yang, J.; Fonseca Rodrigues, M.-T.; Son, S.-B.; Garcia, J. C.; Liu, K.; Gim, J.; Iddir, H.; Abraham, D. P.; Zhang, Z.; Liao, C., Dual-Salt Electrolytes to Effectively Reduce Impedance Rise of High-Nickel Lithium-Ion Batteries. *ACS Applied Materials & Interfaces* **2021,** *13* (34), 40502-40512.
7. Yang, J.; Shkrob, I.; Liu, Q.; Dietz Rago, N. L.; Liu, Y.; Liu, K.; Zhang, Z.; Liao, C., Structural underpinnings of cathode protection by in situ generated lithium oxyfluorophosphates. *Journal of Power Sources* **2019,** *438*, 227039.
8. Peebles, C.; Garcia, J.; Tornheim, A. P.; Sahore, R.; Bareño, J.; Liao, C.; Shkrob, I. A.; Iddir, H. H.; Abraham, D. P., Chemical "Pickling" of Phosphite Additives Mitigates Impedance Rise in Li Ion Batteries. *The Journal of Physical Chemistry C* **2018,** *122* (18), 9811-9824.
9. Garcia, C. P. I. A. S. C. L. D. A. H. I. J. Additives for high voltage lithium ion batteries. 2023.
10. Shui Zhang, S., An unique lithium salt for the improved electrolyte of Li-ion battery. *Electrochemistry Communications* **2006,** *8* (9), 1423-1428.
11. Wang, Y.; Nakamura, S.; Tasaki, K.; Balbuena, P. B., Theoretical Studies To Understand Surface Chemistry on Carbon Anodes for Lithium-Ion Batteries: How Does Vinylene Carbonate Play Its Role as an Electrolyte Additive? *Journal of the American Chemical Society* **2002,** *124* (16), 4408-4421.
12. Zhu, Y.; Li, Y.; Bettge, M.; Abraham, D. P., Positive Electrode Passivation by LiDFOB Electrolyte Additive in High-Capacity Lithium-Ion Cells. *Journal of The Electrochemical Society* **2012,** *159* (12), A2109-A2117.

13. Tornheim, A.; Peebles, C.; Gilbert, J. A.; Sahore, R.; Garcia, J. C.; Bareño, J.; Iddir, H.; Liao, C.; Abraham, D. P., Evaluating electrolyte additives for lithium-ion cells: A new Supplementary Figure of Merit approach. *Journal of Power Sources* **2017,** *365*, 201-209.
14. Xu, K., Nonaqueous Liquid Electrolytes for Lithium-Based Rechargeable Batteries. *Chemical Reviews* **2004,** *104* (10), 4303-4418.
15. Li, W.; Song, B.; Manthiram, A., High-voltage positive electrode materials for lithium-ion batteries. *Chemical Society Reviews* **2017,** *46* (10), 3006-3059.
16. Klein, S.; Haneke, L.; Harte, P.; Stolz, L.; van Wickeren, S.; Borzutzki, K.; Nowak, S.; Winter, M.; Placke, T.; Kasnatscheew, J., Suppressing Electrode Crosstalk and Prolonging Cycle Life in High-Voltage Li Ion Batteries: Pivotal Role of Fluorophosphates in Electrolytes. *ChemElectroChem* **2022,** *9* (13), e202200469.
17. J., E. W. C. S.-S. S. M. B. D. B. N.; Liao;, L. N. T. H. N. S. R. K. R. Z. C.; Persson, K. A., Chemical Reaction Networks Explain Gas Evolution Mechanisms in Mg-Ion Batteries. *J. Am. Chem. Soc.* **2023**.
18. Seeger, M., GAUSSIAN PROCESSES FOR MACHINE LEARNING. *International Journal of Neural Systems* **2004,** *14* (02), 69-106.
19. Gardner, J. R.; Pleiss, G.; Bindel, D.; Weinberger, K. Q.; Wilson, A. G., GPyTorch: blackbox matrix-matrix Gaussian process inference with GPU acceleration. In *Proceedings of the 32nd International Conference on Neural Information Processing Systems*, Curran Associates Inc.: Montréal, Canada, 2018; pp 7587–7597.
20. Landrum, G., Rdkit: Open-Source Cheminformatics Software; GitHub and SourceForge, 2016; Vol. 10, p 3592822.
